# Inflammation fuels bone marrow exhaustion caused by *Samd9l* mutation

**DOI:** 10.1172/JCI164136

**Published:** 2022-11-01

**Authors:** Moonjung Jung

**Affiliations:** Division of Hematology, Department of Medicine, The Johns Hopkins University School of Medicine, Baltimore, Maryland, USA.

## Abstract

Sterile α motif domain–containing 9 (SAMD9) and SAMD9-like (SAMD9L) syndromes are inherited bone marrow failure syndromes known for their frequent development of myelodysplastic syndrome with monosomy 7. In this issue of the *JCI*, Abdelhamed, Thomas, et al. report a mouse model with a hematopoietic cell–specific heterozygous *Samd9l* mutation knockin. This mouse model resembles human disease in many ways, including bone marrow failure and the nonrandom loss of the mutant allele. *Samd9l*-mutant hematopoietic stem progenitor cells showed reduced fitness at baseline, which was further exacerbated by inflammation. TGF-β hyperactivation was found to underlie reduced fitness, which was partially rescued by a TGF-β inhibitor. These findings illustrate the potential role of TGF-β inhibitors in the treatment of SAMD9/SAMD9L syndromes.

## SAMD9/SAMD9L syndromes

Sterile α motif domain–containing 9 (SAMD9) and SAMD9-like (SAMD9L) syndromes are inherited bone marrow (BM) failure syndromes due to mutations in *SAMD9* or *SAMD9L*. The genes are paralogs located on chromosome 7q21 and their function remains elusive but has a growth inhibitory effect. Patients with SAMD9/SAMD9L syndromes have diverse clinical manifestations, including cytopenias, myelodysplastic syndrome (MDS), growth restriction, and immune dysregulation. Previously, *SAMD9* and *SAMD9L* mutations were thought to cause MIRAGE (myelodysplasia, infection, restriction of growth, adrenal hypoplasia, genital phenotypes, and enteropathy) syndrome and ataxia pancytopenia syndrome, respectively (1, 2). But, more recent studies suggest those syndromes can be observed in patients with either gene mutations (3).

SAMD9/SAMD9L syndromes are notorious for their frequent association with monosomy 7, which carries one of the worst prognoses in myeloid malignancies as a single cytogenetic abnormality (4). Because the gain-of-function mutants are severely growth inhibitory, cells that lost the mutant allele (i.e., monosomy 7, uniparental isodisomy 7q, or loss-of-function second-site mutation) have a growth advantage leading to an expansion of clones that lost *SAMD9*/*SAMD9L*-mutation-containing chromosome 7 (5). In fact, patients carrying germline *SAMD9*/*SAMD9L* mutations comprise 8% to 17% of childhood MDS with monosomy 7 (3, 6) and it has been described as familial monosomy 7 syndrome (7). Selection pressure for monosomy 7 underlies a predisposition to MDS and acute myeloid leukemia.

In this issue of the *JCI*, Abdelhamed, Thomas, and colleagues established a mouse model of SAMD9/SAMD9L syndromes. The mice (referred to as *Samd9l-Mut*) possessed a heterozygous *Samd9l* p.W1171R mutation, which is equivalent to the human *SAMD9L* p.W1180R mutation noted in patients with impaired hematopoietic cell function (8).

## Lessons learned from *Samd9l* mouse models

The mouse genome lacks *Samd9* and only has *Samd9l* on chromosome 6, which opens the opportunity to study the function of *Samd9l* without redundancy of *Samd9*.

We have learned from prior *Samd9l* mouse models that (a) *Samd9l* haploinsufficiency (heterozygous knockout) predisposes mice to myeloid malignancies spontaneously, which is even more accelerated by viral infections, and it increases the repopulating capacity of hematopoietic stem progenitor cells (HSPCs) (2); (b) *Samd9l* plays an important role in the degradation of cytokine receptors by endocytosis and endosome fusion with lysosomes, thereby its deficiency increases the availability of cytokine receptors on the cell surface and promotes growth (2, 9); and (c) a gain-of-function *Samd9l* homozygous mutant mouse model develops BM failure, growth retardation, and both homozygous and heterozygous mutant mice show reduced repopulating capacity (9).

In their study, Abdelhamed, Thomas, et al. generated conditional knockin mice that had a skewed myeloid commitment at the expense of decreased lymphoid commitment (8). B cell lymphopenia was the most notable finding in the peripheral blood. Single-cell RNA sequencing (RNA-Seq) further showed a differentiation block in the B cell lineage. *Samd9l*-*Mut* HSPCs also showed decreased colony formation upon replating and were outcompeted in competitive transplantation assays, suggesting decreased overall fitness of *Samd9l*-*Mut* HSPCs, similar to a prior study (2). Interestingly, 4 out of 26 *Samd9l-Mut* mice had a partial loss of chromosome 6 on which mouse *Samd9l* is located, phenocopying nonrandom loss of a mutant allele on chromosome 7 in patients with SAMD9/SAMD9L syndromes. This finding suggests that loss of the *Samd9l*-mutant allele confers a growth advantage in mice, as in humans (8).

## Inflammation and BM failure

Inflammation caused by infection or autoimmune conditions triggers HSCs to transition rapidly from quiescence to active cell cycling with the eventual loss of self-renewal capacity (10–13). Proinflammatory cytokines, including IFN-α and IFN-β, in response to viral infections can cause BM suppression or even aplastic anemia (14–16). This pathology may be due to a direct effect on HSCs or an indirect effect via the niche.

*SAMD9* and *SAMD9L* are IFN-responsive genes and they play an important role in innate immunity against viral infections (17–19). The authors show that one way to directly affect HSCs in the setting of inflammation is through the type I IFN signaling–induced expression of SAMD9L in HSCs. Increased SAMD9L expression favors the elimination of inflamed (or infected) cells by inhibiting their growth. In vitro IFN-α or in vivo polyinosinic/polycytidylic acid (pI:pC) injections, which induce type I IFNs (IFN-α and IFN-β) in vivo, increased the expression of both WT and mutant SAMD9L proteins. However, the treatment substantially increased apoptosis and decreased colony-forming capacity only in *Samd9l*-*Mut* BM cells.

**Figure 1:**
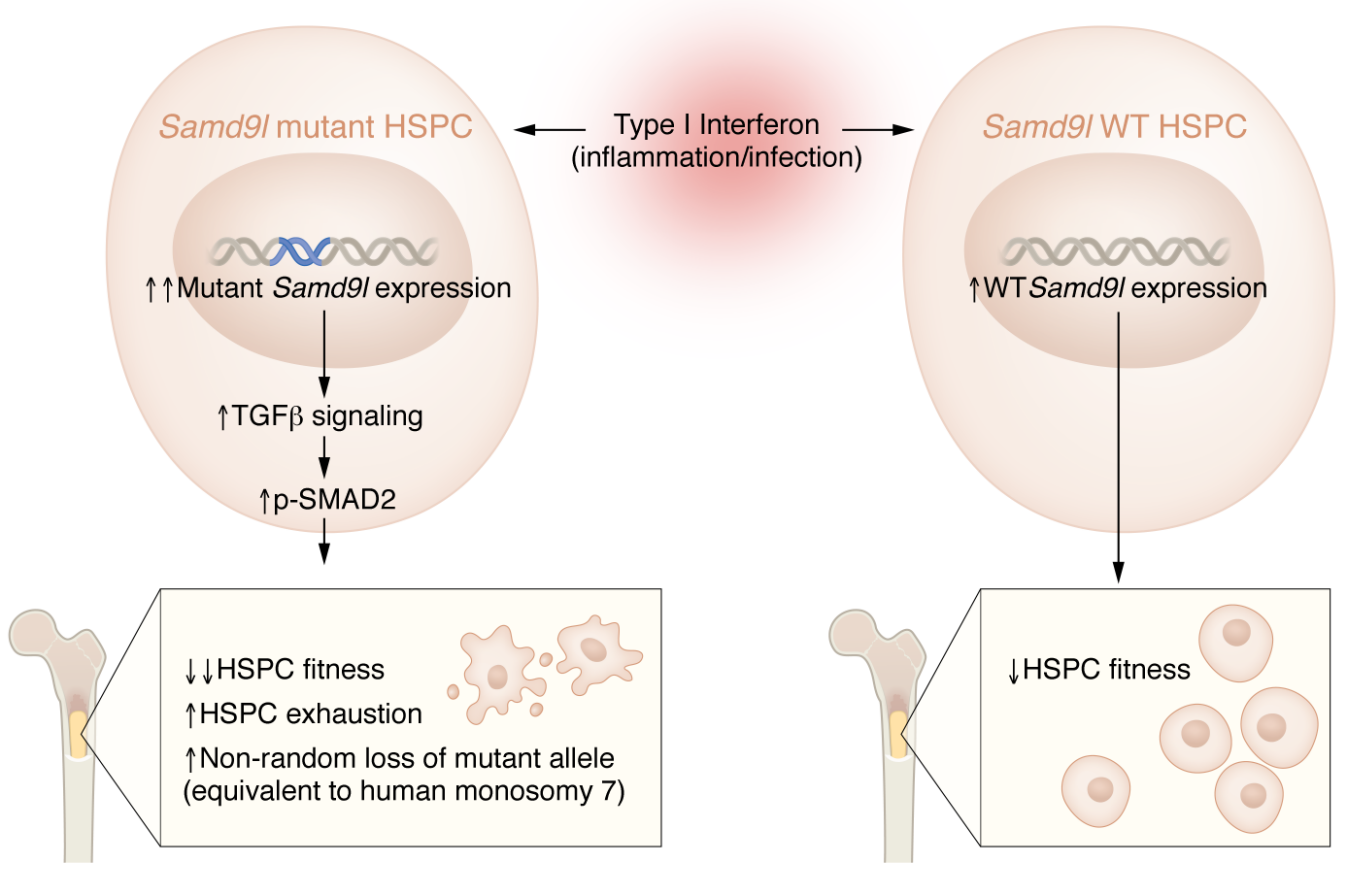
Model for bone marrow exhaustion and monosomy 7 exacerbated by inflammation in SAMD9/SAMD9L syndromes. In a mouse model of SAMD9/SAMD9L syndrome, exposure to type I IFNs increases the expression of both WT and mutant *Samd9l* expression in HSPCs. However, only mutant *Samd9l* leads to upregulation of TGF-β and subsequent p-SMAD2/3 signaling upon inflammatory insults, which further exacerbate already severely decreased HSPC fitness, BM exhaustion, and promote nonrandom loss of mutant allele (equivalent to human monosomy 7) in mutant mice.

Interestingly, inflammation induced by pI:pC reduced the engraftment potential of both *Samd9l-WT* and *Samd9l-Mut*, but it is noteworthy that inflammation further decreased the already reduced engraftment potential and increased apoptosis of *Samd9l*-*Mut* BM cells. Similarly, both *Samd9l-WT* and *Samd9l*-*Mut* mice decreased their lymphocyte counts upon pI:pC challenge, but a greater degree of reduction along with myeloid hyperplasia was observed in *Samd9l*-*Mut* mice. Subsequent RNA-Seq analysis of lineage-negative cKit-positive HSPCs showed that the TGF-β pathway was upregulated in pI:pC-treated *Samd9l*-*Mut* HSCs, which was confirmed by flow cytometry as increased p-SMAD2/3 signaling (Figure 1). This upregulation of p-SMAD2/3 was most notable in B cells, which may explain B cell lymphopenia in *Samd9l-Mut* mice. The TGF-β small molecule inhibitor, SD-208, partially rescued in vitro colony-forming capacity of mutant *Samd9l*, albeit not to the WT levels (8). These findings suggest that TGF-β inhibitors may boost the colony-forming capacity of the *Samd9l-Mut* mouse and patient BM; however, whether the inhibitors can actually reverse BM failure in mice and humans requires future studies.

Interestingly, TGF-β hyperactivation is not specific to SAMD9/SAMD9L syndromes but is also observed in other inherited BM failure syndromes, such as Fanconi anemia (20), Diamond-Blackfan anemia (21, 22), and Shwachman-Diamond syndrome (23). These observations suggest that TGF-β hyperactivation may be a common pathway leading to HSC exhaustion due to various underlying mechanisms.

## Summary and future directions

Abdelhamed, Thomas, et al. generated a *Samd9l* mouse model with a heterozygous human *SAMD9L* mutation equivalent at its endogenous locus. This mouse model phenocopies human disease in that it develops BM failure and nonrandom loss of the mutant allele. The authors further investigated the effects of inflammation using in vitro and in vivo models and showed that inflammation exacerbates apoptosis of lymphocytes, and reduces colony-forming capacity and engraftment potential, particularly in *Samd9l*-*Mut* HSPCs. They discovered that TGF-β hyperactivation was one of the mechanisms underlying reduced HSPC fitness in the setting of inflammation, and a TGF-β inhibitor rescued the colony-forming capacity of mouse and human mutant HSPCs at least partially. It will be critically important to confirm whether TGF-β hyperactivation is present also in patient HSPCs and whether it is only induced with inflammation or also present at a steady state, before moving TGF-β inhibitors to clinical trials. It would also be interesting if TGF-β inhibitors could reverse growth restriction and prolong survival in their mouse model. The work by Abdelhamed, Thomas, et al. opens the possibility of TGF-β inhibitors as therapeutics in this rare disease with no current treatment options other than BM transplant (8).

## Version 1 11/01/2022

Electronic publication
